# The Physiological and Molecular Mechanism of Abscisic Acid in Regulation of Fleshy Fruit Ripening

**DOI:** 10.3389/fpls.2020.619953

**Published:** 2021-01-11

**Authors:** Qian Bai, Yun Huang, Yuanyue Shen

**Affiliations:** ^1^College of Horticulture, China Agricultural University, Beijing, China; ^2^College of Plant Science and Technology, Beijing University of Agriculture, Beijing, China

**Keywords:** fruit ripening, non-climacteric fruit, abscisic acid, ethylene, signaling transduction, review

## Abstract

The ripening of fleshy fruits is coupled with the degradation of both chlorophyll and cell walls, as well as changes in the metabolism of phenylpropanoids, flavonoids, starch/sucrose, and carotenoids. These processes are controlled by phytohormones and other factors, including abscisic acid (ABA), ethylene, auxin, polyamines, sugar, and reactive oxygen species. The ripening of climacteric fruits is controlled by ethylene and non-climacteric fruit ripening is regulated mainly by ABA. Also, ABA and ethylene may interact in both types of fruit ripening. ABA concentrations in fleshy fruits are regulated in response to developmental and environmental cues and are controlled by the relative rates of ABA biosynthesis and catabolism, the former mainly *via* 9-cis-epoxycarotenoid dioxygenases (NCEDs) and β-glucosidases and the latter *via* ABA 8'-hydroxylases (CYP707As) and β-glycosyltransferases. In strawberry fruit ripening, ABA is perceived *via* at least two receptors, Pyrabactin resistance (PYR)/PYR-like (PYL) and putative abscisic acid receptor (ABAR), which are linked separately to the conserved signaling pathway ABA-FaPYR1-FaABIl-FaSnRK2 and the novel signaling pathway ABA-FaABAR-FaRIPK1-FaABI4. Downstream signaling components include important transcription factors, such as AREB (ABA responsive element binding protein)/ABF (ABRE binding factors ABA responsive factor), ethylene response factor (ERF), and V-myb Myeloblastosis viral oncogene homolog (MYB), as well as ripening-related genes. Finally, a comprehensive model of ABA linked to ethylene, sugar, polyamines, auxin and reactive oxygen species in the regulation of strawberry fruit ripening is proposed. Next, new integrated mechanisms, including two ABA signaling pathways, ABA and ethylene signaling pathways, and ABA/ethylene to other phytohormones are interesting and important research topics in ripening, especially in non-climacteric fruits.

## Introduction

Angiosperm fruits can generally be divided into dry or fleshy types. Flowering plants have evolved both conserved and divergent mechanisms for development and maturation of both fleshy and dry fruits (Hershkovitz et al., 2011; Garceau et al., 2017). The molecular mechanisms of dry fruit development and maturation have been revealed through studies of the model plant Arabidopsis (Gazzarrini and Tsai, 2015), while studies of various non-ripening tomato fruit mutants have defined the mechanisms of fleshy fruit ripening (Liu et al., 2015).

Fleshy fruit ripening is mainly divided into two types, climacteric and non-climacteric, based on the presence or absence of a transient peak in respiration rate and ethylene emission. In climacteric fruits, such as tomato and banana, peaks in both respiration and ethylene level occur during ripening; in contrast, no such peaks occur in non-climacteric fruits, such as grape and strawberry, the ripening of which is controlled by abscisic acid (ABA) in an ethylene-independent manner (Kumar et al., 2014; Shen and Rose, 2014). Fruit color break, coinciding with the conversion of chloroplasts into chromoplasts, is a visible sign of ripening in both climacteric and non-climacteric fruits, and is followed by softening and changes in sugars, color, and flavor (Azoulay et al., 2008). During the past decade, studies using strawberry fruit as a model have led to major breakthroughs in understanding the regulation of non-climacteric fruit ripening by ABA and its interaction with other phytohormones. In this review, we focus on the physiological and molecular mechanisms of ABA in regulation of fleshy fruit ripening.

## Central Roles of ABA in Regulation of Non-Climacteric Fruit Ripening

Classical studies of ABA effects on fleshy fruit ripening were done on grape (*Vitis vinifera*) berries and uncovered vital roles for ABA in veraison and ripening processes, including coloration, sugar accumulation, acid decline, and flesh softening (Coombe, 1976; Davies et al., 1997). However, a deeper understanding of ABA action in non-climacteric fruit ripening was gained from a breakthrough study in strawberry (Jia et al., 2011). The importance of ABA has recently been confirmed in many non-climacteric fruits, such as sweet cherry (*Prunus avium*; Tijero et al., 2016; Shen et al., 2017), watermelon (*Citrullus lanatus*; Wang et al., 2017b), blueberry (*Semen trigonellae*; Oh et al., 2018), bilberry (*Vaccinium myrtillus*; Karppinen et al., 2018), jujube (*Ziziphus jujuba*; Kou et al., 2019), litchi (*Litchi chinensis*; Hu et al., 2019), orange (*Citrus sinensis*; Romero et al., 2019), and wolfberry (*Lycium ruthenicum*; Li et al., 2019).

### ABA Perception and Signaling in Strawberry Fruit Ripening Represent a Model of Non-climacteric Ripening

In developing strawberry fruit, ripening is markedly stimulated by exogenous ABA or DMSO (dimethyl sulfoxide, an accelerator of ABA biosynthesis), whereas fluridone (an inhibitor of ABA biosynthesis) significantly inhibits ripening; furthermore, downregulation of either *FaNCED1* (*9-cis-epoxycarotenoid dioxygenase*, a key gene in ABA biosynthesis) or *FaCHLH/ABAR* (*magnesium chelatase H subunit/putative ABA receptor*) inhibits ripening; importantly, exogenous ABA can rescue coloration of *FaNCED1*-RNAi fruit but not of *FaCHLH/ABAR*-RNAi fruit, confirming that ABA is a key signal molecule in the promotion of strawberry ripening (Jia et al., 2011). Kadomura-Ishikawa et al. (2015) identified FaMYB10 as an important transcription factor mediating signal transduction downstream of ABA perception by the ABAR to stimulate anthocyanin biosynthesis during strawberry fruit ripening. ABA and *FaMYB10* are therefore key regulators for strawberry ripening (Kim et al., 2019). In all, ABA homeostasis in cells involves the regulation of both ABA catabolism and ABA biosynthesis by feedback and feedforward loops, which are tightly linked to the repression of *FveCYP707A4a* expression, key to ABA degradation, and promotion of *FveNCED* expression, key to ABA biosynthesis, in response to the onset of strawberry fruit ripening (Liao et al., 2018). Tightly controlled, synergistic regulation of ABA from biosynthesis to signaling is required to meet the requirements of plant development and fruit ripening.

In addition, the importance of ABA in strawberry ripening was also confirmed by the silencing of genes encoding another ABA receptor, FaPYR1 (Pyrabactin Resistance1; Chai et al., 2011), and its interacting protein FaABI1 (type 2C protein phosphatase1; Jia et al., 2013a). A strawberry FERONIA/FER-like receptor kinase, FaMRLK47, negatively regulates ABA signaling and fruit ripening through its interaction with FaABI1 (Jia et al., 2017a). Importantly, the core ABA signaling pathways, Pyrabactin Resistance 1 (PYR1)/PYR1-like (PYL)/Regulatory Component of ABA Receptor (RCAR) – Type 2C Protein Phosphatase (PP2C)-SNF1-Related Protein Kinase2 (SnRK2) are conserved in plant responses to developmental and environmental cues during stress adaptation, flowering, seed germination, and fruit ripening (Li et al., 2011; Cheng et al., 2019; Gupta et al., 2020).

CHLH/ABAR has multiple functions (Liang et al., 2015). For example, in Arabidopsis, CHLH/ABAR regulates various plant developmental processes in different pathways, such as stomatal movement, seed germination, and seedling growth, through ABA-ABAR-WRKY40-ABI5/ABI4 (Shang et al., 2010). To further explore the mechanisms of FaABAR action in strawberry fruit ripening, Hou et al. (2018) used FaABAR as bait in a yeast two-hybrid assay and isolated a strawberry leucine-rich repeat (LRR) receptor-like kinase, red-initial protein kinase 1 (FaRIPK1) that interacts with FaABAR. FaRIPK1 serves as a co-receptor of FaABAR to synergistically regulate strawberry fruit ripening, namely, FaRIPK1 participates in the onset of ripening while FaABAR is responsible for completion of ripening. Importantly, FaABAR binds to ABA with a *K*_d_ (dissociation constant) of 50 μM (Zhang et al., 2017), suggesting that FaABAR is an ABA receptor in strawberry (Jia et al., 2011; Zhang et al., 2017; Hou et al., 2018). Notably, the ABA-ABAR-RIPK1-ABI4 signaling pathway that controls fruit ripening in strawberry (Hou et al., 2018) is distinct from the ABA-ABAR-WRKY40-ABI5/ABI4 pathway in Arabidopsis (Shang et al., 2010).

By contrast, FaPYR1 regulates fruit ripening through the conserved core signaling pathway PYR1-PP2C-SnRK2 (Cheng et al., 2019; Gupta et al., 2020). In addition, among members of FaPYR/PYL and FaPP2C families in strawberry, only FaPYL2/4/9/11 and FaABI1/FaPP2C16 interact with each other, and the interaction of FaPYL2 with FaABI1 might also play a role in strawberry fruit ripening (Hou et al., 2020). Notably, the SnRK2.6/OST1 protein acts as a linker between ABAR and PYR/PYL/RCAR in Arabidopsis guard cells (Liang et al., 2015). Thus, the relationships between ABAR and PYR/PYL/RCAR in strawberry fruit ripening should be established in the future.

### ABA is a Major Player in the Ripening of Various Non-climacteric Fruits

The relationship between ABA and fruit ripening defined in strawberry facilitates studies of ripening in other non-climacteric fruits, such as grape (Villalobos-González et al., 2016), sweet cherry (Shen et al., 2017), watermelon (Wang et al., 2017b), blueberry (Oh et al., 2018), bilberry (Karppinen et al., 2018), jujube (Kou et al., 2019), litchi (Hu et al., 2019), orange (Romero et al., 2019), and wolfberry (Li et al., 2019).

It is previously reported that ABA is a major phytohormone involved in regulating the onset of grape berry ripening and berry skin secondary metabolism (Villalobos-González et al., 2016). ABA positively regulates sweet cherry development, ripening, and quality through the interaction of PacPP2C1 with six PacSnRK2s, including PacSnRK2.2/2.3/2.6 and PacPP2C1-3 (Tijero et al., 2016; Shen et al., 2017). It is also a major player in watermelon fruit ripening (Wang et al., 2017b). While application of ABA to blueberry fruits has no remarkable effect on fruit growth, it stimulates fruit coloration and softening; however, softening is undesirable during harvest and storage (Oh et al., 2018; Chung et al., 2019). Chinese jujube is a non-climacteric fruit, the ripening of which is regulated by ABA (Kou et al., 2019; Zhang et al., 2019). In addition, ABA plays a key role in ripening-related processes including fruit coloration and softening in both bilberry (Karppinen et al., 2018) and orange fruits (Romero et al., 2019). ABA-mediated anthocyanin biosynthesis during wolfberry fruit maturation and coloration involves the transcription factor complex MYB-bHLH-WD40 (Li et al., 2019). Interestingly, recent reports suggest that the grape U-box E3 ubiquitin ligase VlPUB38 negatively regulates berry ripening in an ABA-dependent manner by degradation of abscisic-aldehyde oxidase (VlAAO), the enzyme catalyzing the last step of ABA biosynthesis (Yu et al., 2020). A synergistic interaction between CrNAC036 and CrMYB68 appears to inhibit CrNCED5-mediated ABA biosynthesis at the transcription level in citrus fruit (Zhu et al., 2020).

De-greening (chlorophyll degradation) and coloration (such as anthocyanin biosynthesis) are major physiological changes during fruit ripening. Application of ABA rapidly initiates chlorophyll breakdown in litchi fruit, and peak ABA concentration is concommitant with the subsequent onset of anthocyanin biosynthesis, demonstrating an important role for ABA during ripening (Hu et al., 2019). ABA response element-binding factors (LcABF1/2/3) play a vital role in litchi fruit ripening: *LcABF1* expression increases in parallel with chlorophyll degradation, while *LcABF3* expression rises at the onset of coloration; moreover, LcABF1/2 mediate expression of ABA-responsive genes related to chlorophyll degradation, while LcABF2/3 mediate expression of genes related to anthocyanin biosynthesisin an ABA-dependent manner (Hu et al., 2019). Collectively, ABA is a major player in regulation of the ripening of various non-climacteric fruits.

### Non-climacteric Fruit Ripening is Regulated by ABA Through Multiple Synergistic Mechanisms

In non-climacteric strawberry fruit, ABA and auxin (IAA) are important regulators, functioning together or independently in response to developmental and environmental cues; auxin is involved in receptacle development and ABA participates in fruit ripening. By contrast, ethylene and gibberellins do not play a prominent role in ripening (Medina-Puche et al., 2016). Contents of both IAA and GA4 (gibberellic acid) are highest in small, green strawberry fruit and gradually decrease throughout fruit development; ABA content increases rapidly, coincident with coloration; methyl jasmonate concentration shows no remarkable variation over time, while salicylic acid content gradually increases; and jasmonic acid and ethylene contents are too low to quantify (Kim et al., 2019). Auxin is produced mainly in achenes, while ABA, ethylene, cytokinins and gibberellins are predominantly biosynthesized in receptacles (Gu et al., 2019). Gibberellin delays ripening to some extent, while cytokinins and ethylene appear to be involved in the later stages of ripening, mostly highlighting possible mechanisms of ABA and auxin interaction in the ripening process (Gu et al., 2019). With the onset of fruit ripening in strawberry, the roles of ABA, ethylene, and polyamines are reinforced while those of GA and IAA are weakened (Wang et al., 2017a). Available data from strawberry suggest that regulation of non-climacteric fruit ripening is rather complex.

#### Interaction of ABA With Ethylene

Although ethylene is well known as a key regulator in climacteric fruit ripening (Liu et al., 2015), this gaseous molecule also participates in non-climacteric fruit ripening through its interaction with ABA (Li et al., 2020; Tosetti et al., 2020). In postharvest strawberry fruit, ethylene facilitates ABA accumulation in receptacle tissue but does not affect ABA catabolism (Tosetti et al., 2020). Overexpression of *FveERF* (FvH4_5g04470.1), an ethylene response regulator, activates both acyltransferase (AAT) gene transcription and ester accumulation during strawberry fruit ripening (Li et al., 2020). The promoter regions of *ACS1* from both climacteric “Santa Rosa” plum and its non-climacteric mutant “Sweet Miriam” have only minor differences in sequence; however, *ABI5* (*ABA insensitive5*) expression in “Sweet Miriam” fruit is lower than that in “Santa Rosa” fruit during ripening, suggesting a vital role of ABA in ethylene production (Sadka et al., 2019). A cucumber (*Cucumis sativus*) MADS-box (SHATTERPROOF) protein, CsSHP, participates in fruit maturation through ABA-mediated protein complex associated to CsSEPs (SEPALLATA; Cheng et al., 2020). ABA- and ethylene-related genes are differentially modulated during grape berry ripening by a set of transcription factors, including MADS-box, MYB, NAC, AP2/ERF, bHLH, and ZIP (Wong et al., 2016). Thereby, the interaction of ABA with ethylene plays a vital role in non-climacteric fruit ripening.

#### Interaction of ABA With IAA

Greater accumulation of IAA and ABA in developing achenes than in the receptacle is important for strawberry fruit maturation and ripening, suggesting a complex ripening mechanism regulated by the two hormones (Araújo et al., 2012; Symons et al., 2012). In fact, auxin plays a dominant role in receptacle growth while ABA plays a vital role in ripening, and other hormones function to different degrees with ABA or ethylene (Estrada-Johnson et al., 2017). Some ripening-related genes are co-regulated by IAA and ABA during strawberry fruit development (Castillejo et al., 2004), such as *FaRGlyase1* (rhamnogalacturonate lyase gene; Molina-Hidalgo et al., 2013), *FaSHP* (a C-type MADS-box gene; Daminato et al., 2013), *FaβGal4* (β-galactosidase gene; Paniagua et al., 2016), and *FaNIP1;1* (plasma membrane aquaporin protein gene; Molina-Hidalgo et al., 2015), which are positively regulated by ABA and negatively regulated by auxins during strawberry fruit ripening. Also, annexins FaAnn5 and FaAnn8 might be involved in the regulation of both ABA and IAA during strawberry fruit growth and ripening through calcium signaling (Chen et al., 2016). The receptor-like kinase and ubiquitin ligase respond to both IAA and ABA and may play an important role in crosstalk between the two hormones (Chen et al., 2016). IAA and ABA are therefore key regulators of strawberry fruit maturation and ripening.

Confirmedly, strong “antagonism” between IAA and ethylene and substantial “synergism” between IAA and ABA occur during grape berry development (Ziliotto et al., 2012). At berry pre-ripening, high auxin levels promote seed tissue development, and transcripts of auxin-response-factor genes accumulate in pericarp tissue; at veraison, auxin action is weakened while ABA action is enforced, suggesting a role for a higher IAA/ABA ratio during growth and a lower ratio during ripening (Gouthu and Deluc, 2015). ABA, auxin and ethylene play important roles in the regulation of non-climacteric fruit maturation and ripening *via* a complex network (Corso et al., 2016).

#### Interaction of ABA With Polyamines

Blocking ethylene biosynthesis affects the levels of both ABA and putrescine in melon fruit (Concepción Martínez-Madrid et al., 2002). During strawberry fruit ripening, polyamines (PAs), especially spermine, interact with ABA, IAA, and ethylene in a coordinated manner (Guo et al., 2018). Increased transcription of *NCED3*, key to ABA production, at the onset of strawberry fruit ripening facilitates rapid accumulation of ABA, which inhibits transcription of *FaPAO5* (*polyamine oxidase5*), key to PA degradation, leading to accumulation of spermine and spermidine (Mo et al., 2020). Interestingly, the increase in spermine and spermidine contents triggers expression of SAMDC (SAM decar boxylase), SPDS (spermidine synthase), and SPMS (spermine synthase) genes, key to PA biosynthesis, further accelerating spermine and spermidine accumulation and ripening, suggesting that FaPAO5 regulates spermine and spermidine levels in response to ABA signaling during strawberry fruit ripening (Mo et al., 2020). Thus, interactions between ABA and PA play an important role in the regulation of strawberry fruit ripening.

#### Interaction of ABA With Sugars

Sugars play a central role in fruit ripening and quality because sugar metabolism and accumulation greatly influence taste. Notably, sucrose also serves as a signal, which facilitates strawberry fruit ripening by stimulating ABA production and accumulation (Jia et al., 2011, 2013b). Both ABA and sucrose induce grape berry ripening, with sucrose acting in both ABA-dependent and ABA-independent manners (Jia et al., 2017b). ASR (ABA-, stress-, and ripening-induced protein) regulates strawberry fruit ripening through crosstalk between ABA and sucrose (Jia et al., 2016). Application of sucrose to unripe strawberry fruit promotes ripening by ABA and its derivatives (Siebeneichler et al., 2020). ABA and sucrose accelerate strawberry fruit ripening and induce accumulation of H_2_O_2_, leading to a transient decrease in glycolysis, demonstrating that the interaction of ABA with sucrose affects ripening by inhibiting glycolysis (Luo et al., 2020a). Moreover, FaGAPC2 (cytosolic glyceraldehyde-3-phosphate dehydrogenase)/FaGAPCp1 (plastid glyceraldehyde-3-phosphate dehydrogenase), key glycolytic enzymes, negatively regulate ABA- and sucrose-mediated ripening in strawberry fruit (Luo et al., 2020b). Therefore, the interaction between ABA and sugar plays a pivotal role in strawberry fruit ripening.

## ABA is also Involved in the Regulation of Climacteric Fruit Ripening

Although ethylene is an essential regulator of climacteric tomato fruit ripening (Liu et al., 2015), in which, as has been demonstrated over the past decade, ABA also plays a role. Application of ABA accelerates coloration and ethylene biosynthesis in tomato fruit as well as accumulation of phenolic compounds and emission of volatile aromatics (Wu et al., 2018). A tomato gene key to ABA biosynthesis, *SINCED1*, triggers the onset of tomato fruit ripening (Zhang et al., 2009), and suppression of *SINCED1* expression leads to increases in lycopene and β-carotene contents and in shelf life from 15 to 29 days (Sun et al., 2012a,b). Most PYL ABA receptors interact with SlPP2Cs in an ABA-dependent manner; SlPP2C2/SlPP2C3 interact with SlSnRK2s; SlSnRK2.5 interacts with SlABF2/4; and many core components of ABA signaling respond to ABA (Chen et al., 2016). In addition, downregulating the expression of either *SlUGT75C1* (ABA uridine diphosphate glucosyltransferase gene) or *SlPP2C1* (type 2C protein phosphatase gene) alters tomato fruit ripening (Sun et al., 2017; Zhang et al., 2018). The tomato ABA receptor SlPYL9 positively regulates ABA signal transduction and fruit ripening in an ABA-dependent manner (Kai et al., 2019). Overexpression of *LYCOPENE β-CYCLASE* (*LCYb*) leads to an increase in β-carotene and ABA concentrations, as well as a decrease in ethylene emission, which delays softening and increases shelf life, suggesting that manipulation of β-carotene levels can improve nutritional value and shelf life (Diretto et al., 2020). Similarly, 24 *PYL*, 87 *PP2C*, and 11 *SnRK2* genes have been identified in banana fruit ripening, providing new insights into understanding PYL-PP2C-SnRK2 core signaling (Hu et al., 2017).

The interaction between ABA and ethylene has been studied extensively, contributing to our understanding of the mechanism of ABA involvement in climacteric fruit ripening. ABA may promote ethylene emission and signal transduction by recruiting a set of important genes, including *LeACO1* (*1-amino-cyclopropane-1-carboxylic acid oxidase1*), *LeACS4* (*1-amino-cyclopropane-1-carboxylic acid synthase4*), *GR* (*green-ripe*), and *LeETR6* (*ethylene receptor6*); while ethylene plays an essential role in inducing ABA accumulation at the initiation of ripening, and blocking or removing ethylene leads to low ABA levels (Meyer et al., 2017). Furthermore, a set of transcription factors specific to ethylene production and signaling, including MADS-RIN (MADS-ripening inhibitor), TAGL1 (tomato Agamous-like1), CNR (colorless non-ripening), and NOR (non-ripening), are also ABA responsive, suggesting a crucial function of these transcription factors in ABA-ethylene interactions (Mou et al., 2016). Transient overexpression of *SlAREB1*, a tomato transcription factor gene downstream of ABA signaling, promotes expression of a set of genes including *NOR*, *SlACS2*, *SlACS4*, and *SlACO1*, suggesting that SlAREB1-NOR crosstalk plays a vital role in ABA-mediated ethylene biosynthesis during tomato fruit ripening (Mou et al., 2018).

In addition to tomato, ABA is also involved in ripening of other climacteric fruits. Application of ABA to apple facilitates ethylene emission by AREB/ABF-mediated regulation of *MdACS1/3* and *MdACO1* expression, suggesting a role for ABA in ethylene biosynthesis (Wang et al., 2018). In banana, ABA promotes *MaMADS2* expression and fruit ripening (Yakir et al., 2018). A peach ethylene response transcription factor, PpERF3, positively regulates PpNCED2/3-mediated ABA biosynthesis during fruit ripening (Wang et al., 2019a), while PpERF2 regulates fruit-ripening by inhibiting *PpNCED2/3* expression. PpERF2 binds to the promoters of *PpNCED2*, *PpNCED3*, and *PpPG1* (Wang et al., 2019b).

In all, the ABA-PYL-PP2C-SnRK2 core signaling pathway is also conserved in climacteric fruit. A set of crucial regulators related to the biosynthesis and signaling of both ABA and ethylene, such as NCED, AREB/ABF, ACS, ACO, and ERF, likely play important roles in the interaction of these two hormones, as elucidated by AREB/ABF-mediated *ACS/ACO* expression and ERF-mediated *NCED* expression. MADS-RIN, TAGL1, CNR, and NOR, transcription factors specific to ethylene biosynthesis and signaling, are probably also involved.

## An Integrated, Comprehensive Understanding of ABA in Fleshy Fruit Ripening

The developmental processes of fleshy fruits include cell division and expansion during fruit growth, followed by chlorophyll degradation, cell wall softening, and changes to phenylpropanoid, flavonoid, starch/sucrose, and carotenoid metabolism during ripening. These processes are controlled by phytohormones, most notably ethylene in climacteric fruit ripening and ABA in non-climacteric fruit ripening, as well as ABA-ethylene interactions in both types of fruit (Forlani et al., 2019; García-Gómez et al., 2020; Jiao et al., 2020).

De-greening through loss of chlorophyll is an obvious indicator of fruit ripening, and ethylene and ABA interact with light during chlorophyll degradation (Zhu et al., 2017). In addition, a decrease in water content occurs at the onset of ripening in both climacteric and non-climacteric fruit in an ethylene-independent manner, suggesting that oxidant-induced cell wall remodeling and consequent wall dehydration may trigger stress signaling that initiates fruit ripening (Frenkel and Hartman, 2012). Fruit ripening and senescence are associated with changes in fruit texture, color, aroma, and flavor, processes in which reactive oxygen species play an important role (Kumar et al., 2016). The interaction of auxin with ethylene is important in ripening of both climacteric and non-climacteric fruits (Paul et al., 2012). The APETALA2/ethylene response factor (AP2/ERF) transcription factor is a crucial response regulator in ethylene signaling, which not only modulates biosynthesis of ABA, ethylene, gibberellin, and cytokinin in a feedback manner but also responds to signaling associated with ABA, IAA, jasmonate, and cytokinin (Wang et al., 2015; Gu et al., 2017). The promotion of grape berry ripening by ABA, ethylene, and brassinosteroids, and ripening inhibition by IAA, jasmonic acid, GAs, cytokinins, and PAs reveal complex interactions among signals (Fortes et al., 2015). Notably, polyamines regulate strawberry fruit ripening by ABA, auxin and ethylene (Guo et al., 2018), while FaPAO5 regulates Spm/Spd levels as a signaling during strawberry fruit ripening by integrating multiple signals including Ca^2+^ and GAs (Mo et al., 2020).

**Figure 1:**
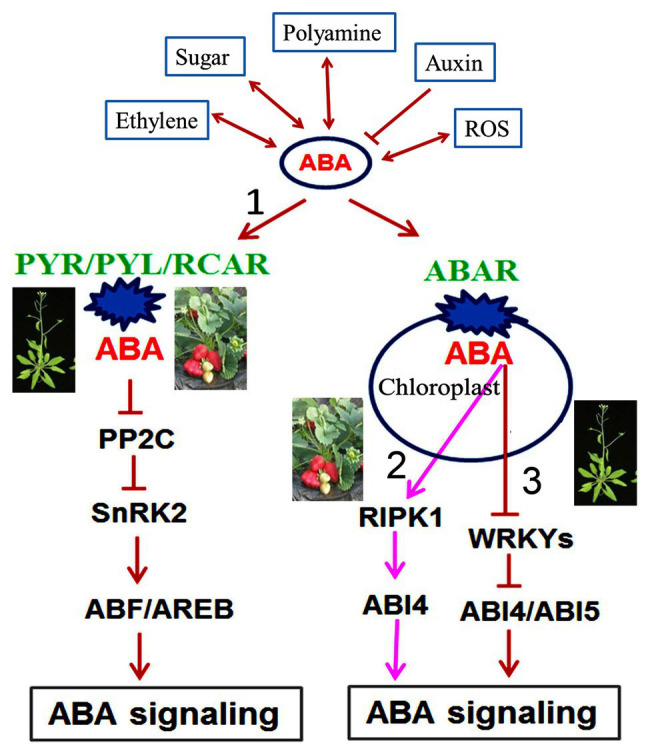
A comprehensive model for ABA in regulating strawberry fruit ripening. The regulation of ABA in strawberry fruit ripening is involved in conserved and novel signaling mechanisms. ABA in fleshy fruits is perceived by its receptors, PYR/PYL/RCAR and ABAR, which initiates signal transduction, including the conserved signaling pathway ABA-PYR/PYL/RCAR-PP2C-SnRK2-ABF/AREB in both Arabidopsis and strawberry (signaling pathway 1) and the novel signaling pathway ABA-FaABAR-FaRIPK1-FaABI4 in strawberry (signaling pathway 2). In strawberry ABA signaling, ABAR/FaRIPK1 serves as a receptor complex, which is distinct from the Arabidopsis ABA-ABAR-WRKY40-ABI4/ABI5 signaling pathway (signaling pathway 3). ABA interacts with several synergistically-regulated molecules such as ethylene, sugar, polyamines, auxin, and reactive oxygen species (ROS) during strawberry fruit ripening. The symbols (→, ⟞, and ↔) represent promotion, inhibition, and cooperation, respectively. ABA, abscisic acid; ABAR, putative abscisic acid receptor; PYR, Pyrabactin resistance; PYL, PYR-like; RCAR, regulatory components of ABA receptors; PP2C, Type 2C protein phosphatase; SnRK2, SNF1-related protein kinases2; ABF, ABRE binding factors ABA responsive factors; AREB, ABA responsive element binding protein; ROS, reactive oxygen species; RIPK1, red-initial protein kinase1; ABI, abscisic acid-insensitive; WRKY, tryptophan-arginine-lysine-tyrosine.

Finally, we propose an integrative model of ABA regulation of fleshy fruit ripening (Figure 1). In response to developmental and environmental cues, ABA levels in fleshy fruits are controlled by ABA biosynthesis and catabolism; the former mainly through NCEDs and β-glucosidases and the latter through CYP707As and β-glycosyltransferases. ABA molecules in fleshy fruit are perceived *via* the ABA receptors PYR/PYL and ABAR, which are linked to the conserved signaling pathway ABA-FaPYR1-FaABIl-FaSnRK2 and the novel signaling pathway ABA-FaABAR-FaRIPK1-FaABI4 in strawberry, respectively. In the conserved ABA signaling pathway, ABA levels increase at the onset of fruit ripening; consequently, the accumulated ABA binds to its receptor FaPYR1 to form a FaPYR1-FaABIl complex, releasing the inhibition of protein kinase FaSnRK2 and in turn activating an ABA signaling cascade, which promotes fruit ripening. This pathway constitutes the conserved mechanism of ABA action in plants. In the novel ABA signaling pathway, ABA binding to the receptor FaABAR/FaRIPK1 complex activates the transcription factor FaABI4, which triggers ABA signal transduction, eventually promoting fruit ripening. This signaling pathway is different from the Arabidopsis ABA-ABAR-WRKY40 signaling pathway, thus constituting a novel ABA signaling pathway during strawberry fruit ripening.

## Conclusions

Fruit ripening is controlled by phytohormones, with ripening of climacteric fruits controlled by ethylene and that of non-climacteric fruits regulated mainly by ABA, as well as by ABA-ethylene interplay in the two types of fruit ripening. ABA participates in the ripening of both non-climacteric and climacteric fruit. The core signaling pathway ABA-PYL-PP2C-SnRK2 is conserved, and a series of crucial regulators including NCED, AREB/ABF, ACS, ACO, and ERF may play important roles in ABA-ethylene interaction, particularly AREB/ABF-mediated *ACS/ACO* expression and ERF-mediated *NCED* expression. ABA levels in fleshy fruit are controlled through a balance between ABA biosynthesis and catabolism involving key enzymes, including NCEDs, β-glucosidases, CYP707As, and β-glycosyltransferases. ABA in fleshy fruits is perceived by at least two ABA receptors, PYR/PYL and ABAR, which are linked separately to the conserved signaling pathway ABA-FaPYR1-FaABIl-FaSnRK2 and the novel signaling pathway ABA-FaABAR-FaRIPK1-FaABI4 in strawberry fruit ripening. This model provides novel insights into the role of ABA in ripening, at least in strawberry, a model plant for non-climacteric-type fruits. Next, new integrated mechanisms, including two ABA signaling pathways, ABA and ethylene signaling pathways, and ABA/ethylene to other phytohormones and regulators, are interesting and important research topics in ripening, especially in non-climacteric fruits.

## Author Contributions

YS, YH, and QB wrote the review. QB collected references. YS and YH designed the model and revised the review. All authors contributed to the article and approved the submitted version.

### Conflict of Interest

The authors declare that the research was conducted in the absence of any commercial or financial relationships that could be construed as a potential conflict of interest.
